# Early-stage olfactory bulbectomy induces hyperlocomotion with increased astrocyte and microglial density in the prefrontal cortex of male rats

**DOI:** 10.1007/s00429-026-03139-z

**Published:** 2026-06-13

**Authors:** Mario Alberto Bautista-Carro, Diana Moroni-González, Julia Flores-Tochihuitl, Gonzalo Flores, Julio César Morales-Medina

**Affiliations:** 1 Centro de Investigación y de Estudios Avanzados del Instituto Politécnico Nacional, Unidad Tlaxcala, Autopista San Martín Texmelucan. Tlaxcala, Km. 10,5 San Felipe Ixtacuixtla, Tlaxcala de Xicohténcatl, 90128 México; 2 Departamento de Fisiología, Biofísica y Neurociencias, Centro de Investigación y de Estudios Avanzados del Instituto Politécnico Nacional, Av. IPN 2508, San Pedro Zacatenco, Ciudad de México, 07360 México; 3 Laboratorio Multidisciplinario, Facultad de Estomatología, Benemérita Universidad Autónoma de Puebla, Av. 31 Pte. 1304, Los Volcanes, Heroica Puebla de Zaragoza, Puebla, 72410 México; 4 Instituto de Fisiología, Benemérita Universidad Autónoma de Puebla, Prol. 14 Sur 6301, Ciudad Universitaria, Heroica Puebla de Zaragoza, 72592 México

**Keywords:** Astrocytes, Depression, Open field, Olfactory bulbectomy, Prefrontal cortex

## Abstract

Major depressive disorder (MDD) severely affects people’s productivity and quality of life. Olfactory bulbectomy (OBX) yields reproducible behavioral changes resembling agitated depression. Since most studies examine models after the depressive phenotype is fully established, this work examines mood disorder and glial structure during the early period following OBX, aiming to identify initial circuit adaptations that precede the MDD development. Male Wistar rats were assigned to OBX or SHAM groups. Experiments were conducted exclusively in male animals to reduce hormonal variability, which may limit generalizability. One week after surgery, exploratory behavior was assessed using the open-field test. Subsequently, brain samples of prefrontal cortex (Cg1), hippocampus (CA1), and basolateral amygdala were collected. Immunohistochemical stains were performed to evidence astrocytes (GFAP) and microglia (Iba-1), followed by cell counts and morphometric analyses. OBX rats showed increased total and peripheral distance traveled, with no differences in center distance, rearing, or grooming. Region-specific analyses revealed a significant increase in astrocytes and microglia in the PFC, with no significant changes in Sholl- derived morphological parameters. In the CA1, a significant increase in the length of fourth-order microglial branching was observed. Finally, no significant changes were observed in the BLA across any analyzed parameter. These results show a phenotype that resembles agitated depression and demonstrates differential region-specific glial susceptibility. The PFC is the area that shows the first changes in cell number, without morphological changes, suggesting small adaptations that can later give rise to the manifestations described above by our working group.

**Supplementary Information:**

The online version contains supplementary material available at 10.1007/s00429-026-03139-z.

## Introduction

Major depressive disorder (MDD) is a disease with a high prevalence in the population, which has positioned itself as one of the leading causes of disability and, therefore, a significant public health problem, directly impacting quality of life, productivity, and life expectancy. According to World Health Organization (WHO) data, 5.7% of adults suffer from this disease, and it is believed to result from the complex interaction of various social, physiological, and biological factors (GBD 2019 Mental Disorders Collaborators 2022; WHO, 2025). Among the most accepted theories of the pathophysiology of MDD are alterations of neuronal systems and dysfunction of monoaminergic systems; however, given the clinical heterogeneity, the immune system has been adopted as a fundamental component in the development of this mental disorder. In line with this, the role of glia as a regulator of brain function has been recognized in recent decades (Wohleb et al. 2016; Malhi and Mann 2018; Scuderi et al. 2021).

The brain structures in which glial alterations associated with major depressive behaviors have been observed include the prefrontal cortex (PFC), amygdala, and hippocampus, where stress conditions can induce glial changes associated with structural, metabolic, and functional remodeling (Miguel-Hidalgo et al. 2000; Si et al. 2004; Torres-Platas et al. 2011; Cobb et al. 2016). Importantly, these areas are part of the fronto-limbic circuitry that regulates emotional processing, stress responses, and cognitive integration (Arnsten 2009; Roozendaal et al. 2009; McKlveen et al. 2016; Morales-Medina et al. 2017). Notably, they are anatomically and functionally connected to the olfactory bulbs through direct and indirect projections (Coppola and Parrish Waters 2021). The PFC receives multisensory and limbic inputs that integrate olfactory information into behavioral responses (Arnsten 2009; Godsil et al. 2013). The basolateral amygdala (BLA) is involved in assigning emotional salience to olfactory and contextual stimuli (Roozendaal et al. 2009; McEwen et al. 2016), and the hippocampus, particularly, the CA1 region, participates in contextual memory and spatial processing linked to olfactory cues (Song and Leonard 2005). Early glial alterations, especially in astrocytes and microglia, are believed to precede neuronal remodeling and to contribute causally to depressive behaviors (Kreisel et al. 2014; Pekny et al. 2016; Wohleb et al. 2018).

There is a well-established association between olfactory function and MDD (Lombion-Pouthier et al. 2006; Rodrigues et al. 2023). Recent findings indicating astrocytic dysfunction in the olfactory bulbs of samples obtained from depressed individuals further support a critical role of olfactory bulb pathology in the pathophysiology of MDD (Rahimian et al. 2024). Indeed, olfactory bulbectomy (OBX) has the advantages of being a widely studied and reproducible animal model of depression- related behavior. This model allows the analysis of phenotypes characterized by psychomotor agitation (Kelly et al. 1997; Morales-Medina et al. 2017; Coppola and Parrish Waters 2021). Unlike other stress-based models, OBX induces a sustained disruption of fronto-limbic circuits, which also shows previously characterized neurochemical and neuroinflammatory changes (Morales-Medina et al. 2017). In particular, the OBX model reproduces specific aspects of clinical subtypes associated with agitated depression, such as hyperlocomotion, which is why it is helpful in the study of early neuronal and glial mechanisms and adaptations associated with these mental disorders (Machado et al. 2020). Previously, we showed that after 4 weeks of OBX, there is a decrease in cell proliferation in the dentate gyrus and rearrangement of pyramidal neurons in CA1 dorsal hippocampus (Morales-Medina et al. 2013a, b). Recently, our group observed an increase in the number of glial fibrillary acidic protein (GFAP)-positive astrocytes, c-Fos-positive cells and nitric oxide levels in the PFC (Bautista-Carro et al. 2026). These results highlight an essential connection between astrocyte dysfunction and neuronal dysregulation in this model associated with MDD (Bautista-Carro et al. 2026). Notably, most studies in OBX have focused on evaluations performed in intermediate or late stages after surgery, when the depressive phenotype is considered fully established (Song and Leonard 2005; Morales-Medina et al. 2017; Coppola and Parrish Waters 2021). In contrast, behavioral and neurobiological events that occur during the early post-injury phases have received considerably less attention, despite emerging evidence indicating that initial alterations may condition the subsequent progression (Almeida et al. 2017). Understanding these early events is essential to identify critical windows of vulnerability and potential therapeutic targets (Wohleb et al. 2016). Therefore, the present study aimed to evaluate the novelty in the open field test (OFT) and glial alterations in the Cg1 region of the PFC, BLA, and hippocampus (CA1) induced by OBX at an early point, 7 days after surgery. In line with the research conducted by our group, this work seeks to provide evidence on the initial events that may precede the consolidation of the classic depressive phenotype, contributing to a more precise understanding of the temporal and regional progression of the OBX model.

## Methods

### Animals

Adult male Wistar rats (*n* = 20, 2–3 months old) from the Centro de Investigación en Reproducción Animal (Cinvestav, Unidad Tlaxcala, México) were used for all experiments. All the animals were randomly assigned to the SHAM or OBX surgery group. The animals were housed in groups of three to four rats with controlled temperature (20 ± 2 °C), a 12 h light–dark cycle, with unrestricted access to food and water. After surgery, they received post-surgical care; in all procedures, efforts were made to reduce or avoid animal suffering. National Mexican guidelines for laboratory animal care (NOM-062-ZOO-1999), the ARRIVE recommendations (Kilkenny et al. 2010), the Guide of the Care and Use of Laboratory Animals, and institutional ethical standards were followed. Animals showing signs of distress during the study were evaluated by a veterinarian and euthanized if necessary. Only rats with complete olfactory bulb removal and intact frontal cortex (verified postmortem) were included in the analyses. The sample sizes used in this study are consistent with previous reports that employ the OBX model and similar morphological analyses (Bautista-Carro et al. 2026; Galindo-Paredes et al. 2023; Kelly and Leonard 1997; Machado et al. 2020; Morales-Medina et al. 2012, 2013a, b).

### Olfactory bulbectomy surgery

OBX was performed following established protocols (Bautista-Carro et al. 2026; Galindo-Paredes et al. 2023). Rats were anesthetized intraperitoneally using a ketamine–xylazine mixture prepared in sterile saline solution (0.75 ml, 0.25 ml, 5 ml, respectively) at a dose of 0.125 ml/20 g body weight. After shaving and disinfecting the skull with benzalkonium chloride and iodopovidone, a midline incision (1 cm) exposed the frontal bone. Using a stereotaxic drill, two circular openings (approximately 2 mm diameter) were made anterior to bregma (8 mm) and lateral to the midline (2 mm). The olfactory bulbs were aspirated using a fine cannula attached to a vacuum system, with care taken not to disturb adjacent prefrontal regions. SHAM animals underwent the same procedure except for bulb removal. Hemostatic sponge (Spongostan, Ethicon, Inc., USA) was used to control bleeding, and the incision was sutured and covered with iodopovidone. Post-surgery, animals received 4mL of Hartmann’s solution subcutaneously (PiSA, México) and recovered for 1 week. For more details, see (10.17504/protocols.io.81wgbwqjngpk/v1).

### Open field test

The test was conducted in a black square arena (85 × 85 × 60 cm) with even lighting at 300 lux. On the day of the test, the rats were accustomed to the laboratory for 1 h, and subsequently, each animal was placed in the center of the arena to explore freely for 5 min. Once this was concluded, the rat was removed from the field and placed in a new box separate from its group. The arena was cleaned after each animal with 70% alcohol to eliminate olfactory cues on both the base and the walls. As additional controls, during the rat examination, the experimenter was kept out of the laboratory, and all tests were performed between 7:00 am and 12:00 pm to avoid circadian influences. For more details, see (10.17504/protocols.io.yxmvmbqo5g3p/v1). The tests were videotaped from the center of the field, 2 m from the ground, with a Canon camcorder (model Vixia HF R70) with a 2.8–89.6 mm lens, saving the videos in AVI format with 25 fps.

Using this test, one of the most frequent behaviors in OBX rats is an increase in total locomotion, which has been interpreted as a deficit in adaptation to a new environment (Bautista-Carro et al. 2026; Kelly et al. 1997; Leonard and Tuite 1981; Morales-Medina et al. 2013a, b). Furthermore, an increase in the frequency of vertical activity (rearing and grooming) has been interpreted as anxiety-like behavior (Homberg et al. 2002; Kalueff et al. 2016; Lever et al. 2006; Sturman et al. 2018).

Analysis of vertical activity was carried out manually (Morales-Medina et al. 2012), while horizontal activity was analyzed using the Mouse Behavioral Analysis Toolbox (MouBeAT) program in Fiji/ImageJ (Schneider et al. 2012; Bello-Arroyo et al. 2018). The total distance traveled, both peripheral and central, constituted the exploratory activity (Bello-Arroyo et al. 2018). Around 9000 frames contemplated in the Tiff stack were analyzed, using a computer with Intel Core i5-10300 H CPU @ 2.50 GHz, 2.50 GHz; RAM: 24.0 GB; Operating system: 64-bit, Windows 11 Home Single Language.

### Immunohistochemistry

After OFT, the rats were anesthetized with ketamine/xylazine cocktail and underwent transcardiac perfusion with 300 ml of fresh, cold phosphate-buffered saline (1x PBS, pH 7.4), followed by 300 ml of 4% paraformaldehyde (PFA), for review see (Bautista-Carro et al. 2026; Hernández-Echeagaray et al. 2025). After, the brain was removed and postfixed in 4% PFA for 48 h. Coronal slices (40 μm) of brain were taken on a vibratome (Leica, VT1000S microsystem, USA), and using Paxinos and Watson coordinates, sequential slices for Cg1 region of the PFC (+ 3.70 to + 1.70 mm bregma), CA1 of the dorsal hippocampus, and BLA (-2.3 to -3.30 mm bregma) were collected in four-row wells with 1x PBS. At the end of sectioning, all slices were cryoprotected with glycerol: ethylene glycol: 1x PBS (3:3:4) and stored at -20 °C until use.

For immunostaining on day 1, cryoprotect solution was washed out with 1x PBS (4 times / 5 minutes). Next, endogenous peroxidase was inactivated with H_2_O_2_ (1.5% in 1x PBS) for 5 minutes. Consequently, the slices were rinsed in 1x PBS (2 times / 5 min) and then incubated in the blocking solution [1x PBS + 0.3% Triton X-100 + 3% serum [normal rabbit serum for GFAP or normal goat serum for ionized calcium binding adaptor molecule 1 (Iba1)] for 2 hours. Finally, primary antibody incubation was performed using GFAP primary antibodies for astrocytes [goat anti-GFAP (ab53554, abcam, 1:500)] or for microglia [rabbit anti-Iba1 (cell signaling, 1:1000)] in diluent antiserum [1x PBS + 0.3 Triton X-100 + 1% normal rabbit serum (for GFAP) or 1% normal goat serum (for Iba1)] for 24 h at room temperature. On day 2, antibodies were washed 3 times with 1x PBS for 5 min. Secondary antibody incubation was done for 1 hour, using for GFAP: anti-goat [1:250] (VECTASTAIN, Elite, ABC-HRP Kit, Peroxidase, PK-6105, CA, USA) or for Iba1: anti-rabbit [1:500] (VECTASTAIN, Elite, ABC-HRP Kit, Peroxidase, PK-6101, CA, USA) dissolved in diluent antisera for GFAP or Iba1, respectively, at room temperature. The non-conjugated secondary antibody was washed with 1x PBS (3 times/ 5 minutes). Subsequently, avidin-biotin complex was used to incubate sections for 1 hour at room temperature, and once finalized the sections were revealed with 3–3’-diaminobenzidine for 2:30 min (Peroxidase substrate kit DAB, SK-4100, Vector Laboratories Inc., CA, USA). Tissues were washed in 1x PBS two times for 5 min and once in distilled water for 5 min before putting the slices on gelatinized slides for posterior analysis. For more details, see (10.17504/protocols.io.3byl46de8go5/v1).

Using an optical microscope (DM 2000 microscope, Leica Microsystems, USA) equipped with a camera lucida, an observer outside the group reconstructed the three-dimensional glial structure into a two-dimensional plane of five cells in each cerebral hemisphere (ten cells per region). The Sholl analysis was performed for glial cells according to previously published methods (Galindo-Paredes et al. 2023; Bautista-Carro et al. 2026). On each glial cell, a transparent grid with concentric rings spaced 5 μm apart was placed, and the number of intersections per ring was quantified. The total number and the total length of glial branches was calculated in each order from the center of the cell body to the end of the glial branch by multiplying the total number of intersections of each ring by 5 μm, respectively. Bilaterally, five coronal sections were manually counted for each brain region at 40x magnification (DM 2000 microscope, Leica Microsystems, USA) to quantify astrocytes or microglia (Bautista-Carro et al. 2026).

### Statistical analysis

Behavioral test results of OFT (total distance travelled, distance travelled in outer and center region, number of rearing and grooming), total number of astrocytes and microglia, and total branch length were analyzed by two-tailed unpaired t-test. Glial Sholl analysis (branch order length, radial distance, and number of cells per section) was analyzed by two-way ANOVA, followed by Sidak test for *post hoc* comparisons. The Shapiro-Wilk test was used to determine normality. Since the variances between groups were unequal, comparisons were performed using Welch’s t-test. A statistically significant difference was considered at *p* < 0.05. The results were represented as the mean ± standard error for all experiments. The software GraphPad Prism 8 (GraphPad Software Inc., San Diego, CA, USA) was used to analyze data.

## Results

### Experimental design

**Fig. 1:**
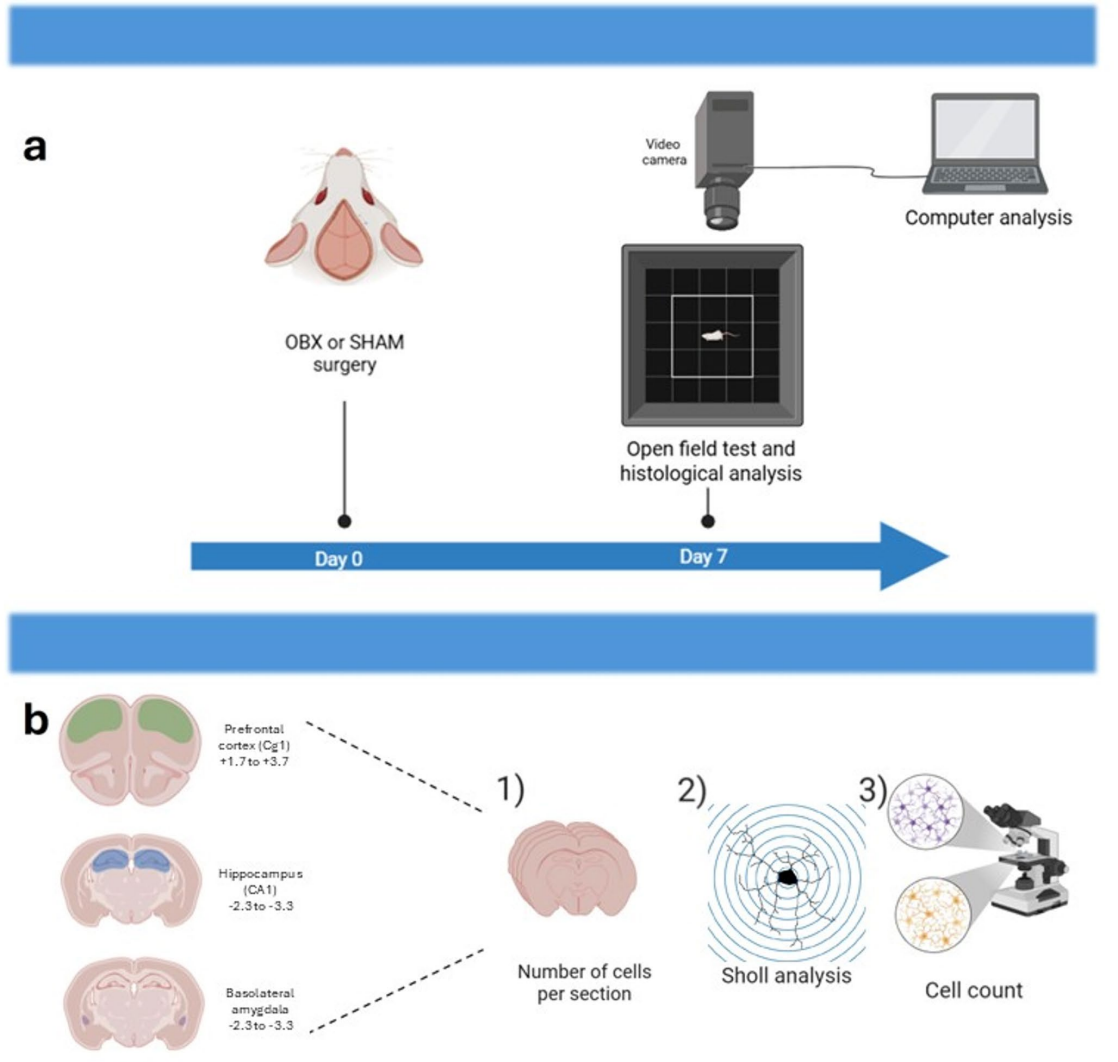
Experimental design. **a** Schematic representation of time–lapse events occurring in the SHAM and OBX groups, 7 days after the surgery. After the open field test, euthanasia was performed, and the brain was obtained for histological examination. **b** Analysis performed on the SHAM and OBX groups included: (1) number of cells per section, (2) Sholl circles are used to determine the arborization, total branching length, and length for branching order on coronal slices of Cg1 of the PFC (+3.7 to +1.7 mm), CA1 (-2.3 to -3.3 mm), and BLA (-2.3 to -3.3 mm). Representative drawing of microglia created using a camera lucida, and (3) total cell count

To analyze the OBX effect on the frontal-limbic-hippocampal circuit, two distinct groups were generated: OBX and SHAM, as described in the methodology. In all of them, behavioral tests and characterization of microglia and astrocytes were performed as shown in Fig. 1.

### Behavioral characterization

**Fig. 2:**
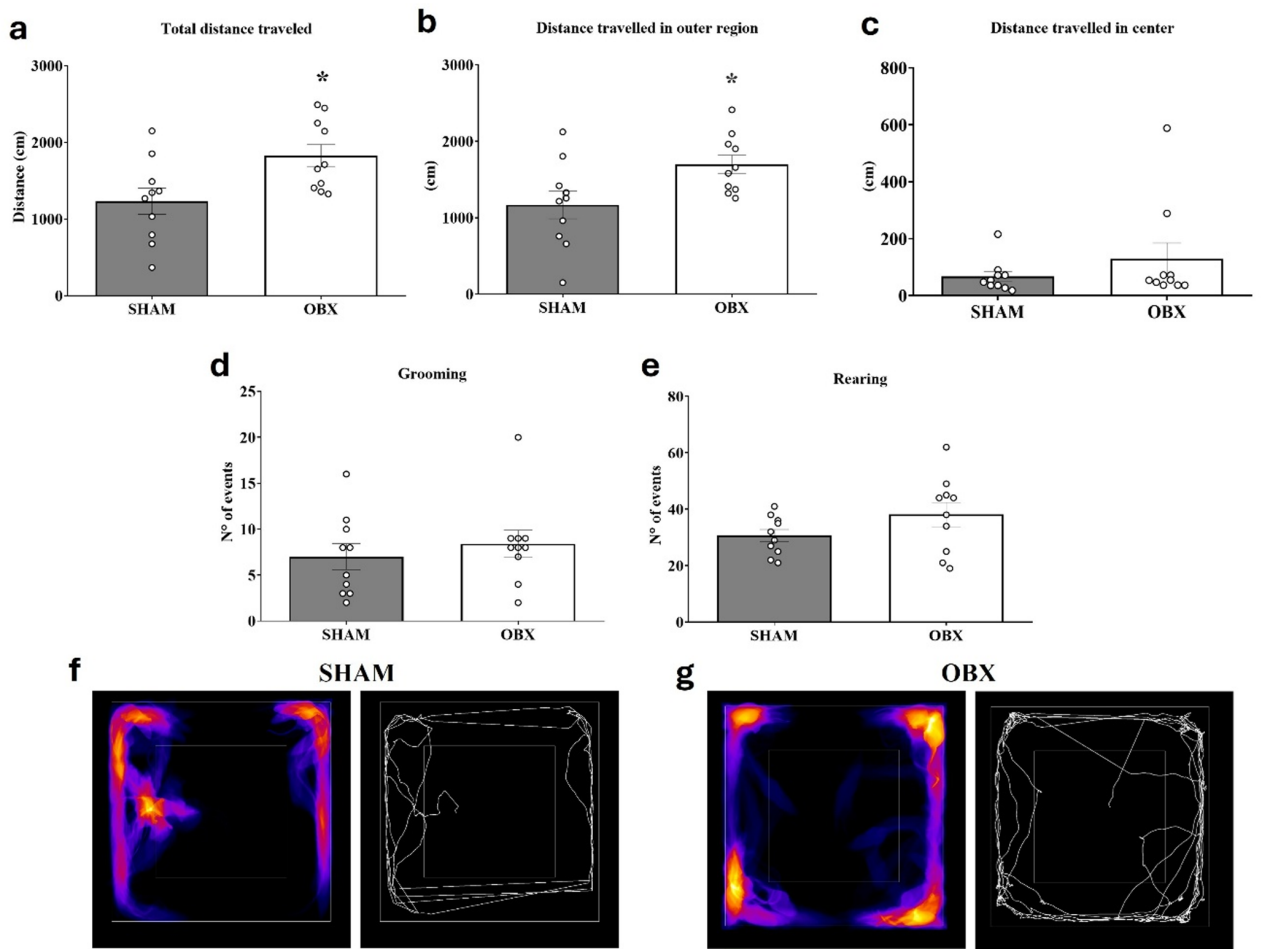
Behavioral analysis during the open field test. Behavioral performance of SHAM and OBX rats. **a** Total distance traveled, **b** Distance traveled in outer region, **c** Distance traveled in center, **d** Grooming events, **e** Rearing events, and heat map and linear trajectory of **f** SHAM and **g** OBX group. Behavioral comparisons were performed using unpaired Welch’s t-tests. *Shows a significant statistical difference at *p* < 0.05. Data are mean ± SEM (*n* = 10)

OBX animals show marked alterations in locomotor behavior compared with SHAM controls. In the OFT, hyperactivity were evaluated by total distance traveled. As shown in Fig. 2a, the OBX group showed a 48% increase in distance traveled (unpaired Welch’s t-test, *t*(17.60) = -2.633, *p* = 0.0171; Cohen´s d = 1.18), at the expense of the distance traveled in the external region (2.5% up) Fig. 2b (unpaired Welch’s t-test *t*(15.78) = -2.439, *p* = 0.0270; Cohen´s *d* = 1.09). In contrast, distance traveled in center (2c), grooming (2d), and rearing (2e) behaviors did not differ between groups (Supplementary Table 1; non-significant results). However, these measures showed a mild tendency toward increased exploratory events in OBX rats. Altogether, these behavioral changes show a profile of increased locomotion with no anxiety-like behavior in OBX animals, providing the functional context in which the specific glial alterations described below develop. Given the behavioral alterations observed in the OBX model, we next examined region-specific cellular changes in astrocytes and microglia, assessing CA1, the PFC, and the BLA.

### Glial morphology in the PFC

**Fig. 3:**
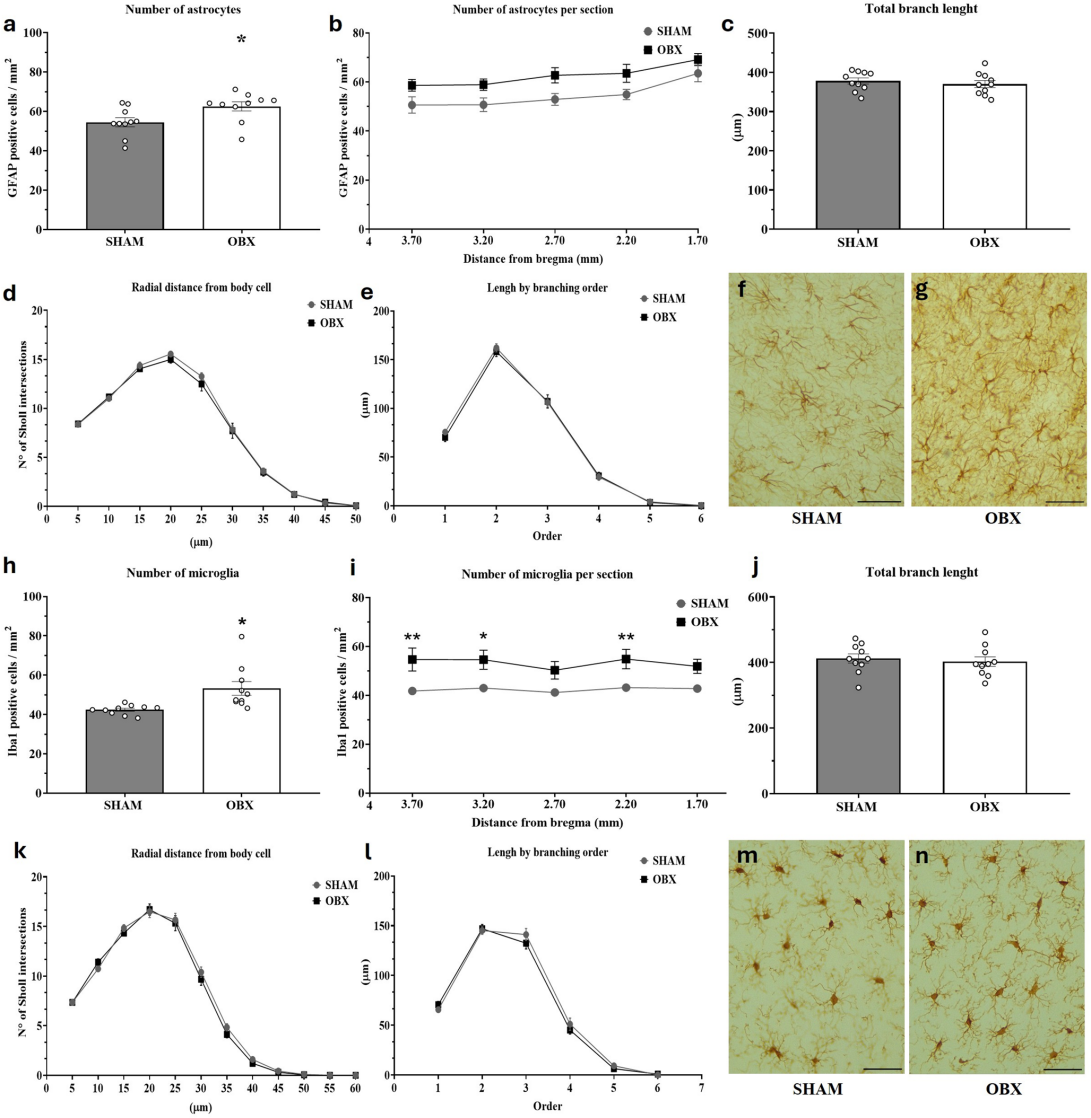
Glial alterations in the Prefrontal Cortex. Astrocytic and microglial profiles of SHAM and OBX rats. **a–g** GFAP-positive astrocytes, **h-n** Iba1-positive microglia: **a**, **h** cell number, **b**, **i** distance from bregma distribution. Sholl analyses: **c**, **j** total branch length, **d**, **k** arborization of branches, **e**, **l** length for each branching order. Representative microphotographs of astrocytes (**f**, **g**) and microglia (**m**, **n**). The figures were taken at 400x magnification, and the scale bar equals 50 μm. Sholl and branching order analyses were evaluated using two-way ANOVA; unpaired Welch’s t-tests were used for cell number and total branch length. *Indicates statistical significance at *p* < 0.05. Data are represented as the mean ± SEM (*n* = 10)

In the PFC, OBX rats exhibited a 15% increase in the number of GFAP-positive astrocytes (Fig. 3a) (unpaired Welch’s t-test, *t*(18) = -2.463, *p* = 0.0241; Cohen´s *d* = 1.10). When examining their distribution across rostro-caudal levels (Fig. 3b), the increase was consistent and not restricted to any specific distance from bregma [two–way ANOVA, group F (1,90) = 19.93, *p* = < 0.0001, η²*p* = 0.18; distance F (4,90) = 5.594, *p* = 0.0005, η²*p* = 0.20; interaction F (4,90) = 0.1448, *p* = 0.09649, η²*p* = 0.006]. Despite this higher astrocyte density, Sholl analysis revealed that total astrocytic branch length remained unchanged (Fig. 3c). Likewise, neither the radial complexity (Fig. 3d) nor the length per branching order (Fig. 3e) showed detectable modifications, indicating that OBX induced astrocytic proliferation without altering morphological complexity. (Supplementary Table 2; non-significant results).

Microglial responses in the PFC displayed a slightly different profile. There was significant 26% increase in the total number of Iba1-positive microglia (Fig. 3h) (unpaired Welch’s t-test, *t*(9.876) = -3.047, *p* = 0.0125; Cohen´s *d* = 1.36). At specific bregma distances, OBX Iba1 positive cells exceeded SHAM [two-way ANOVA, group F (1, 90) = 36.53, *p* < 0.001, η²*p* = 0.29; distance F (4, 90) = 0.4421, *p* = 0.7779, η²*p* = 0.019; interaction F (4, 90) = 0.1791, *p* = 0.9487, η²*p* = 0.008] (Fig. 3i). At distances of 3.70 (*p* = 0.0093), 3.20 (*p* = 0.0245) and 2.20 mm (*p* = 0.0228) from bregma, Sidak’s *post hoc* analysis showed significant statistical increases (around of 20–25%) of microglia in OBX vs. SHAM. Nevertheless, as with astrocytes, microglial morphology remained stable: total branch length did not differ (Fig. 3j), and both radial distribution (Fig. 3k) and order-specific branch length (Fig. 3l) showed overlapping patterns between groups. (Supplementary Table 2; non-significant results).

The relatively pronounced astrocytic changes observed in the PFC (Fig. 3a–e) were consistent with the microglial profile (Fig. 3h–l). Given the strong functional connectivity between the PFC and hippocampus (Godsil et al. 2013; Young et al. 2024) and previous reports of hippocampal susceptibility in OBX models (Morales-Medina et al. 2013a, b; Morales-Medina et al. 2013a, 2017), we next examined the CA1 region of the hippocampus.

### Glial morphology in the Hippocampal CA1 region

**Fig. 4:**
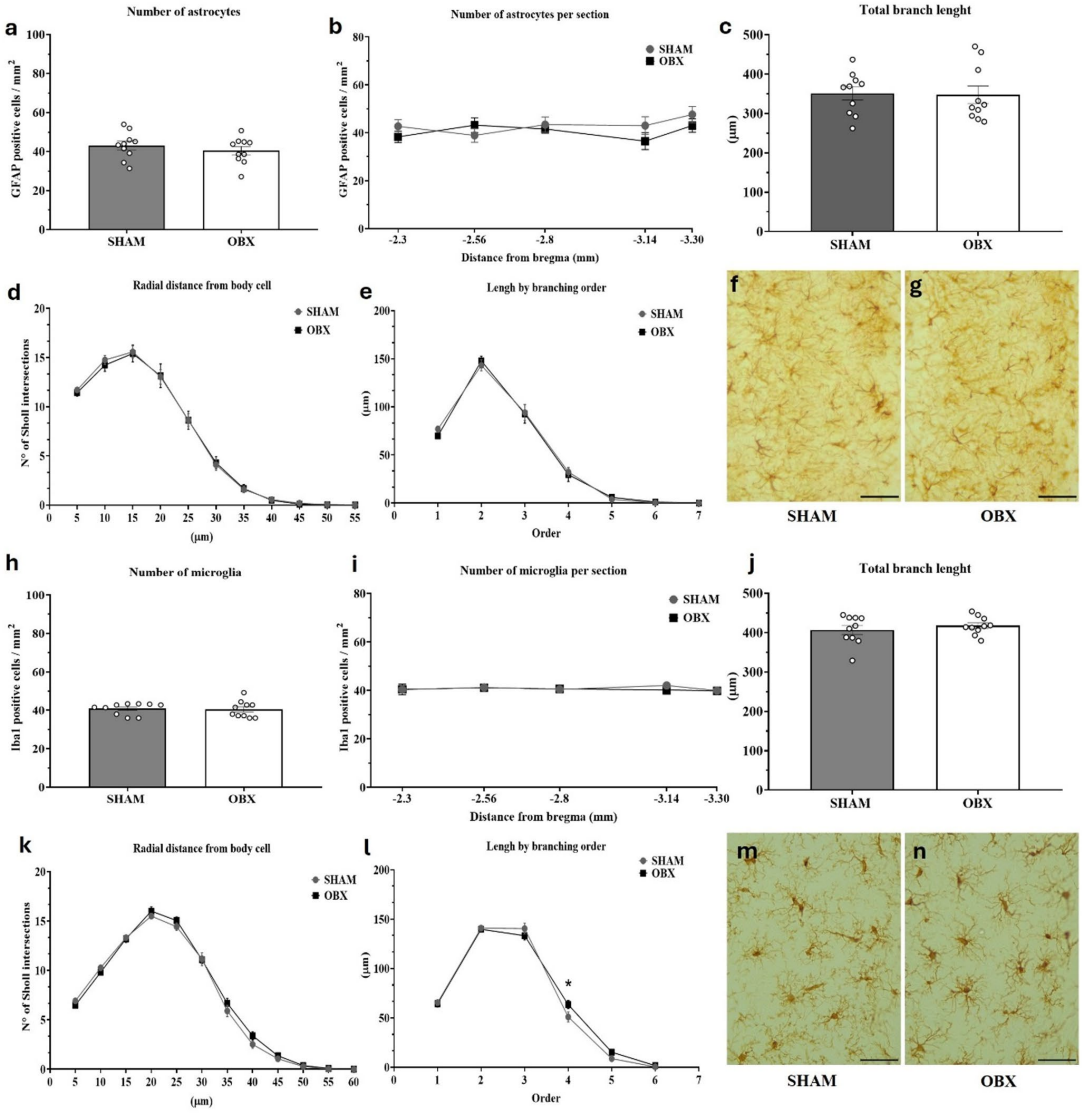
Glial alterations in the CA1 Hippocampus. Astrocytic and microglial profiles of SHAM and OBX rats. **a–g** GFAP-positive astrocytes, **h-n** Iba1-positive microglia: **a**, **h** cell number, **b**, **i** distance from bregma distribution. Sholl analyses: **c**, **j** total branch length, **d**, **k**arborization of branches, **e**, **l** length for each branching order. Representative microphotographs of astrocytes (**f**, **g**) and microglia (**m**, **n**). The figures were taken at 400x magnification, and the scale bar equals 50 μm. Sholl and branching order analyses were evaluated using two-way ANOVA; unpaired Welch’s t-tests were used for cell number and total branch length. * Indicates statistical significance at *p* < 0.05. Data are the mean ± SEM (*n* = 10)

In CA1, the number of GFAP-positive astrocytes did not differ between groups (Fig. 4a). Their rostro-caudal distribution (Fig. 4b) and the total branch length measured from morphological reconstructions (Fig. 4c) remained comparable. Similarly, radial complexity (Fig. 4d) and branching-order length (Fig. 4e) were not modified by OBX. Microglial density in CA1 was also stable, with no differences in the number of Iba1-positive cells (Fig. 4h) or in their distribution across bregma positions (Fig. 4i). Although total branch length and radial complexity remained unaltered (Fig. 4j and k), (Supplementary Table 3); the branching-order profile (Fig. 4l) revealed a significant increase in an intermediate order [two- way ANOVA group F (1, 108) = 0.9093, *p* = 0.3424, η²*p* = 0.008; order F (5, 108) = 662.8, *p* < 0.0001, η²*p* = 0.97; interaction F (5, 108) = 2.191, *p* = 0.0605, η²*p* = 0.09]. In order 4, Sidak’s *post hoc* analysis shows a significant statistical 24% increase in OBX rats compared with SHAM (*p* = 0.0469; Cohen´s *d* = 1.21). Given the absence of a significant interaction and the fact that this increase was not accompanied by changes in total cell count or radial complexity, it should be interpreted with caution and considered an exploratory effect specific to the order, rather than evidence of a generalized difference between the groups. Because CA1 showed minimal glial alterations (mostly limited to Fig. 4l) and given the behavioral phenotype observed, it was relevant to explore whether a region was more directly linked to emotional processing. The BLA displayed no glial adjustments associated with OBX.

### Glial morphology in the BLA

**Fig. 5:**
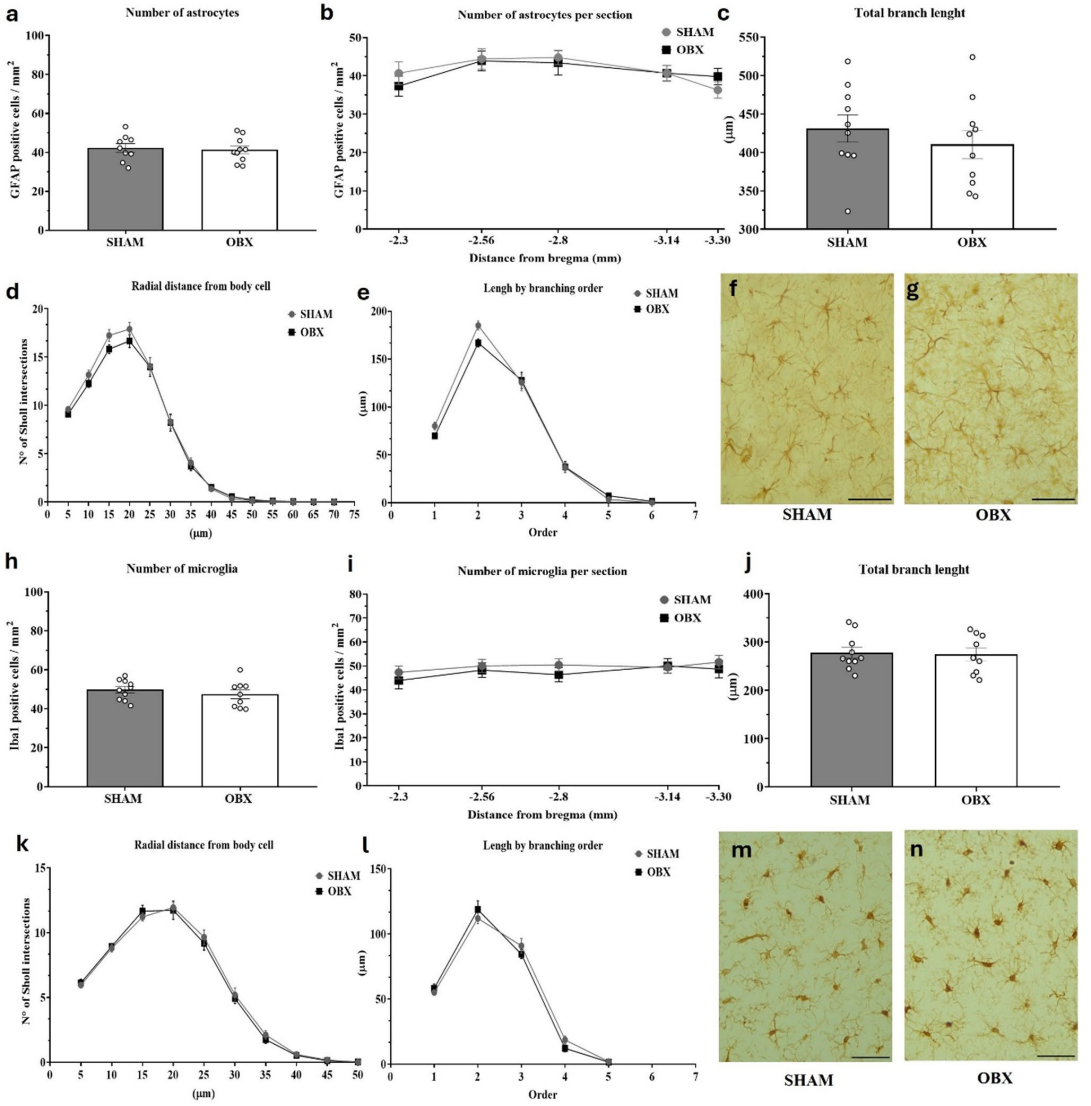
Glial effect in the basolateral amygdala. Astrocytes and microglia of SHAM and OBX rats. **a–g** GFAP-positive astrocytes, **h-n** Iba1-positive microglia: **a**, **h** cell number, **b**, **i** distance from bregma distribution. Sholl analyses: **c**, **j** total branch length, **d**, **k** arborization of branches, **e**, **l** length for each branching order. Representative microphotographs of astrocytes (**f**, **g**) and microglia (**m**, **n**). The figures were taken at 400x magnification, and the scale bar equals 50 μm. Sholl and branching order analyses were evaluated using two-way ANOVA; unpaired Welch’s t-tests were used for cell number and total branch length. * Indicates statistical significance at *p* < 0.05. Data are represented as the mean ± SEM (*n* = 10 − 9)

In the case of astrocytes in BLA, the total number of GFAP-positive cells did not differ significantly between groups (Fig. 5a). Nevertheless, OBX animals displayed a slight downward trend in astrocyte number, which was also apparent in the variation across distance from bregma levels (Fig. 5b). This modest reduction did not reach statistical significance. Similarly, the total length of astrocytic processes showed a mild tendency to decrease in the OBX group (Fig. 5c). This pattern was consistent with the Sholl analysis, where radial complexity (Fig. 5d) and branching-order profiles (Fig. 5e) followed trajectories closely aligned with SHAM animals, yet with a slight overall reduction in amplitude in OBX rats. (Supplementary Table 4; non-significant results). Microglial measurements were similar to those of astrocytes. The number of Iba1-positive cells remained comparable between groups (Fig. 5h), and no group differences emerged across distance from bregma levels (Fig. 5i). Total branch length (Fig. 5j), radial arborization (Fig. 5k), and branching order (Fig. 5l) showed highly similar patterns across conditions. (Supplementary Table 4; non-significant results). Overall, the BLA data reveal a stable glial landscape, with only slight tendencies toward reduced astrocyte number and process length that fall short of statistical significance. These findings contrast with the clearer glial changes observed in the PFC and support a region-specific pattern in the OBX model, in which the amygdala appears relatively resistant to early glial remodeling under the conditions tested.

## Discussion

The present study evaluated early effects of OBX, focusing on seven days after surgery to highlighting first adaptations in the fronto-limbic circuits (Fig. 1). Traditionally, our group and others have characterized the OBX model based on behavioral assessments carried out two to four weeks after the injury, when the depressive phenotype is considered fully established (Morales-Medina et al. 2013a, b; Linge et al. 2013; Bautista-Carro et al. 2026). Our results indicate that OBX induced hyperlocomotion shortly after injury. This behavioral change was associated with increased glial density in the PFC. Together, these findings provide insight into the early behavioral profile of the OBX model.

### Early locomotion alterations in the OBX model

At seven days post-OBX, animals showed increased locomotion, without modifications in center exploration. This is associated with OBX-induced hyperlocomotion, described in the literature (Coppola and Parrish Waters 2021; Kelly et al., 1997; Morales-Medina et al. 2017). Rodents typically balance exploration between central and peripheral areas. Preference for the periphery reflects defensive behavior in novel environments (Walsh and Cummins 1976; Ennaceur 2014). In OBX, this pattern has been linked to loss of olfactory input and altered sensory integration (Coppola and Parrish Waters 2021). The effects of OBX disrupted behavior, including hyperlocomotion, emerge early and do not depend exclusively on later neuroplastic reorganization (Coppola and Parrish Waters 2021; Nedogreeva et al. 2020). However, other studies report increased peripheral and central locomotion at 2 weeks post-OBX, associated with behavioral disinhibition (Machado et al. 2020; Song and Leonard 2005). Despite increased locomotion, rearing and grooming remained unchanged. While rearing reflects vertical exploration, related to environmental assessment (Lever et al. 2006; Sturman et al. 2018; Walsh and Cummins 1976), grooming is associated with self-care and emotional regulation (Kalueff and Tuohimaa 2005; Smolinsky et al. 2009). Their stability suggests that increased locomotion reflects a shift in exploratory strategy, rather than global exploration. This dissociation has been reported in models of agitated depression and psychomotor activation, where locomotion increases without changes in rearing or grooming (Machado et al. 2020; Sturman et al. 2018). In OBX, this pattern of increased locomotion without grooming or rearing effects is common in early phases or under moderate stress (Song and Leonard 2005; Machado et al. 2020). These findings support the idea that distinct behavioral domains are regulated by partially independent circuits. Overall, at seven days post-OBX, the model exhibits locomotor hyperreactivity, predominantly in the periphery, without modification of other behaviors. This state may represent a critical phase before more profound structural and functional alterations.

### Glial remodeling in the PFC after OBX

The PFC showed the most robust and consistent glial alterations consistent with its role in behavioral control, emotional regulation, and cognitive-affective integration. At seven days post-OBX, rats showed a significant increase in GFAP-positive astrocytes in the Cg1 region of the PFC, with homogeneous rostro-caudal distribution and no changes in morphological complexity or process length. This pattern suggests early proliferative astrogliosis, rather than reactive hypertrophy. In early phases of brain damage or stress, astrocytes can increase in number without developing complex morphological changes typical of advanced reactive states (Fu et al. 2024; Pekny and Pekna 2014; Sofroniew and Vinters 2010). In this regard, the absence of modifications in Sholl analysis and branch length supports induces an initial, potentially reversible astrocyte response preceding deeper structural reorganization. The PFC’s vulnerability in the OBX model is well documented as previous studies reported alterations in monoaminergic neurotransmission, increased oxidative stress and inflammation markers, and changes in the expression of genes related to synaptic plasticity after OBX (González-Arias and Perea 2025; Machado et al. 2020; Morales-Medina et al. 2017). In addition, *post-mortem* studies in major depression also show increased GFAP-positive astrocyte density in prefrontal regions, particularly in states associated with psychomotor agitation (Rajkowska et al. 1999; Rajkowska and Stockmeier 2013).

Microglial density also increased in the PFC without morphological changes. This suggests early activation characterized by proliferation rather than full reactive transformation. Microglia can respond progressively showing increases in the number and expression of activation markers, followed later by more evident morphological changes (Helmut et al. 2011; Nimmerjahn et al. 2005). Previously, an increase in proinflammatory cytokines, such as TNF-α and IL-1β, were described in frontal and hippocampal regions after OBX (Morales-Medina et al. 2017). The temporal overlap between hyperlocomotion and glial activation in the PFC (Fig. 3) suggests a functional relationship between them. The PFC is critical for behavioral inhibition, decision-making, and emotional modulation in response to novel or stressful stimuli. Early alterations in glial homeostasis may impair these processes, promoting motor disinhibition or psychomotor agitation (Arnsten 2009; McKlveen et al. 2016). Astrocytes and microglia regulate glutamatergic neurotransmission, redox balance, and neuromodulators such as nitric oxide and purines, all involved in OBX and depression (Araque et al. 2014; Machado et al. 2020). In particular, prefrontal glial activation can alter synaptic efficiency and connectivity, even without morphological changes, affecting behavioral output (Rajkowska and Stockmeier 2013). Thus, early glial responses in the PFC may contribute to the initial behavioral phenotype of the OBX model, preceding later structural and synaptic changes described in later stages.

### Limited glial response and structural preservation in the CA1 hippocampus in early stages of the OBX model

Unlike the PFC, the CA1 region of the dorsal hippocampus showed remarkable glial stability seven days after OBX. This set of results suggests that, in this early time lapse, the astrocyte population of CA1 remains structurally intact. This finding is relevant, as the hippocampus is highly vulnerable in the OBX model, showing structural, neurochemical, and functional alterations at later stages (Morales-Medina et al. 2017; Song and Leonard 2005). However, several studies have indicated that these alterations tend to emerge progressively, becoming more evident from the second- or fourth week post-injury, suggesting a temporally staggered response across brain regions (Almeida et al. 2017; Morales-Medina et al. 2017). In agreement with astrocyte stability, the total number of Iba1-positive microglia in CA1 did not differ between groups. Detailed analysis of the branching order profile revealed a significant increase in an intermediate branching order in OBX rats (Fig. 4l). This change was isolated and was not accompanied by alterations in other morphological parameters, potentially indicative of microglia in an early sensitization (Helmut et al. 2011; Nimmerjahn et al. 2005; Perry and Holmes 2014). Indeed, microglia can exhibit subtle modifications in arborization before developing more pronounced reactive morphological phenotypes, especially in mild inflammation or chronic stress (Helmut et al. 2011). In the hippocampus, these initial alterations have been associated with changes in synaptic surveillance and plasticity modulation, without necessarily implying immediate neurotoxicity (Nimmerjahn et al. 2005). This pattern is consistent with models proposing that hippocampal dysfunction in OBX is not an immediate but a secondary consequence of prolonged alterations in fronto-limbic circuits, sustained stress, and chronic inflammation (Morales-Medina et al. 2017; Rajkowska and Stockmeier 2013). In this sense, glial preservation in CA1 may partly explain why the early behavioral phenotype in the open field shows hyperlocomotion and novelty reactivity without cognitive or spatial memory deficits typically associated with hippocampal dysfunction (Song and Leonard 2005). In addition, clinical studies show that hippocampal glial alterations in MDD are more subtle and variable than in prefrontal regions, especially in early stages or psychomotor subtypes (Enache et al. 2019; Rajkowska et al. 1999). Thus, the results support the idea that OBX progression follows a regional sequence of glial events, which the PFC as an early vulnerable node, followed later by the hippocampus.

### Early glial resistance of the BLA after OBX

Unlike the PFC and in accordance with CA1 stability, the BLA showed a preserved profile at seven days post-OBX. The amygdala, particularly the basolateral subregion, shows distinct temporal dynamics in response to neurobiological insults, with structural and glial changes, emerging later or depending on stressor type (McEwen et al. 2016; Roozendaal et al. 2009). In chronic stress models, the BLA may exhibit early neuronal hyperexcitability without overt glial alterations, with microglial and astrocytic changes emerging after prolonged stress (Munshi et al. 2020). This dissociation between neuronal and glial responses has been reported in stress paradigms with delayed microglial remodeling in the amygdala (Tynan et al. 2010; Zhang et al. 2025). Consistent with astrocyte findings, the microglial population in the BLA remained stable at 7 days post-OBX. The absence of microglial activation in BLA contrasts with the PFC and supports a regional hierarchy of susceptibility to OBX. Previous studies show that amygdala microglia can remain in a baseline state during early stress, becoming evident only after chronic inflammation or persistent synaptic changes (Tynan et al. 2010; Zhang et al. 2025). Additionally, the BLA exhibits region-specific neuroimmune mechanisms that constrain early inflammatory responses. Microglial activation in this region is tightly controlled and often requires prolonged stress exposure, contributing to the stability of emotional circuits during early stages (Bollinger and Wohleb 2019; Tynan et al. 2010; Wohleb et al. 2016).

The glial resistance observed in the BLA is relevant when integrated with the early behavioral phenotype in the OFT. Although the amygdala plays a central role in fear and anxiety, the absence of glial changes suggests that early peripheral hyperlocomotion after OBX may not depend on amygdala dysfunction, but on higher control areas such as the PFC. This aligns with neurocircuitry models proposing that prefrontal dysfunction precedes and modulates amygdala reactivity, while structural changes in the amygdala emerge in later stages or under sustained stress (Arnsten 2009). In humans, neuroimaging and *post-mortem* studies show that amygdala glial and volumetric alterations are less consistent and more dependent on clinical subtype and disease duration than prefrontal changes (Enache et al. 2019; Rajkowska and Stockmeier 2013). The early increase in astrocyte and microglial density in the PFC at 7 days post-OBX likely reflects initial glial priming or proliferation triggered by remote neuroinflammatory and neuroendocrine signals (Calcia et al. 2016). At this early stage, glial activation was predominantly numerical rather than morphological, suggesting that structural remodeling requires sustained stress and emerges later as we have previously observed (Bautista-Carro et al. 2026).

### Limitations

Although the present study provides relevant evidence of early behavioral and glial alterations induced by OBX, several limitations should be acknowledged. First, while detailed quantitative and morphometric analyses of astrocytes and microglia were performed using Sholl-based approaches, the study did not incorporate functional or molecular markers of glial activity, such as inflammatory cytokine profiles, metabolic state indicators, or gene expression analyses. Given that glial cells can undergo substantial functional modulation in the absence of overt morphological remodeling, particularly during early or low-grade activation states, it is possible that functional alterations occurred that were not captured by the morphometric methodologies employed.

In addition, the experimental design was restricted to a single sex, which limits the generalizability of the findings. Accumulating evidence indicates that sex-specific differences exist in stress responsivity, glial activation dynamics, and vulnerability to affective disorders, with males and females often exhibiting distinct neuroimmune trajectories and behavioral phenotypes. Consequently, it will be important to conduct future studies that incorporate sex as a biological variable to determine the extent to which the early glial and behavioral changes observed here are conserved or diverge between sexes. Given the exploratory and hypothesis-generating nature of several analyses in this study, we did not apply a formal multiple-comparisons correction across all tests.

## Conclusion

The present study reveals that the behavioral phenotype induced by OBX is characterized primarily by reactivity to novelty at an early stage accompanied by a regionally differentiated pattern of glial alterations. At the same time, glial changes exhibit selective activation in the PFC, with increases in density in astrocytes and microglia, in the absence of complex morphological remodeling. This pattern suggests that, in the early phases of the OBX model, vulnerability is not homogeneous but follows a hierarchical sequence in which the PFC emerges as a particularly sensitive initial node.

This regional dissociation supports the notion that the early behavioral alterations observed after OBX do not depend on generalized limbic dysfunction but instead on an initial involvement of prefrontal circuits responsible for inhibitory control and modulation of emotional responses. Early prefrontal glial activation could alter synaptic and neuromodulator homeostasis, facilitating the expression of states of psychomotor hyperreactivity. Taken together, these findings support an early-sequential model of the OBX, in which prefrontal dysfunction precedes and potentially precipitates subsequent limbic alterations, which is essential for establishing the temporal progression of the model and linking it to affective states characterized by psychomotor agitation.

## Supplementary Information

Below is the link to the electronic supplementary material.

Supplementary Material 1

## Data Availability

All raw data generated and analyzed for this article are not publicly available due to intellectual property reasons. But they are available through the corresponding author upon reasonable request.

## Declarations

### Statements

This study was conducted in independent cohorts, and no data from the same animals or tissues have been published.

## References

[CR2] Araque A, Carmignoto G, Haydon PG et al (2014) Gliotransmitters travel in time and space. Neuron 81:728–739. 10.1016/j.neuron.2014.02.007 24559669 10.1016/j.neuron.2014.02.007 PMC4107238

[CR3] Arnsten AFT (2009) Stress signalling pathways that impair prefrontal cortex structure and function. Nat Rev Neurosci 10:6 10:410–422 10.1038/nrn2648 10.1038/nrn2648 PMC2907136 19455173

[CR4] Bautista-Carro MA, Sánchez-Teoyotl P, Juárez-Serrano D, Iannitti T, Díaz A, Flores G, Morales-Medina JC (2026) Olfactory bulbectomy induces neurobiological alterations in the prefrontal cortex and hyperlocomotion in male rats. PLoS ONE 21(1):e0339028. 10.1371/journal.pone.0339028 41592094 10.1371/journal.pone.0339028 PMC12843528

[CR5] Bello-Arroyo E, Roque H, Marcos A et al (2018) MouBeAT: A new and open toolbox for guided analysis of behavioral tests in mice. Front Behav Neurosci 12:409231. 10.3389/FNBEH.2018.00201/BIBTEX 10.3389/fnbeh.2018.00201 PMC6137138 30245618

[CR6] Bollinger JL, Wohleb ES (2019) The formative role of microglia in stress-induced synaptic deficits and associated behavioral consequences. Neurosci Lett 711. 10.1016/j.neulet.2019.134369 10.1016/j.neulet.2019.134369 PMC9875737 31422099

[CR7] Calcia MA, Bonsall DR, Bloomfield PS et al (2016) Stress and neuroinflammation: a systematic review of the effects of stress on microglia and the implications for mental illness. Psychopharmacology 233:1637. 10.1007/S00213-016-4218-9 26847047 10.1007/s00213-016-4218-9 PMC4828495

[CR8] Cobb JA, O’Neill K, Milner J et al (2016) Density of GFAP-immunoreactive astrocytes is decreased in left hippocampi in major depressive disorder. Neuroscience 316:209–220. 10.1016/j.neuroscience.2015.12.044 26742791 10.1016/j.neuroscience.2015.12.044 PMC4836620

[CR9] Coppola DM, Parrish Waters R (2021) The olfactory bulbectomy disease model: A Re-evaluation. Physiol Behav. 10.1016/j.physbeh.2021.113548. 240:34371022 10.1016/j.physbeh.2021.113548

[CR1] de Almeida RF, Ganzella M, Machado DG et al (2017) Olfactory bulbectomy in mice triggers transient and long-lasting behavioral impairments and biochemical hippocampal disturbances. Prog Neuropsychopharmacol Biol Psychiatry 76:1–11. 10.1016/J.PNPBP.2017.02.013 28223107 10.1016/j.pnpbp.2017.02.013

[CR10] Enache D, Pariante CM, Mondelli V (2019) Markers of central inflammation in major depressive disorder: A systematic review and meta-analysis of studies examining cerebrospinal fluid, positron emission tomography and post-mortem brain tissue. Brain Behav Immun 81:24–40. 10.1016/J.BBI.2019.06.015 31195092 10.1016/j.bbi.2019.06.015

[CR11] Ennaceur A (2014) Tests of unconditioned anxiety - Pitfalls and disappointments. Physiol Behav 135:55–71. 10.1016/j.physbeh.2014.05.032 24910138 10.1016/j.physbeh.2014.05.032

[CR12] Fu YW, Jin SY, Li JT et al (2024) Mature astrocytes as source for astrocyte repopulation after deletion in the medial prefrontal cortex: Implications for depression. Glia 72:1646–1662. 10.1002/GLIA.24573 38801194 10.1002/glia.24573

[CR13] Galindo-Paredes G, Flores G, Morales-Medina JC (2023) Olfactory bulbectomy induces nociceptive alterations associated with gliosis in male rats. IBRO Neurosci Rep 14:494–506. 10.1016/J.IBNEUR.2023.05.006 37388490 10.1016/j.ibneur.2023.05.006 PMC10300455

[CR14] GBD 2019 Mental Disorders Collaborators (2022) Global, regional, and national burden of 12 mental disorders in 204 countries and territories, 1990–2019: a systematic analysis for the Global Burden of Disease Study 2019. Lancet Psychiatry 9:137–150. 10.1016/S2215-0366(21)00395-3 35026139 10.1016/S2215-0366(21)00395-3 PMC8776563

[CR15] Godsil BP, Kiss JP, Spedding M, Jay TM (2013) The hippocampal-prefrontal pathway: The weak link in psychiatric disorders? Eur Neuropsychopharmacol 23:1165–1181. 10.1016/j.euroneuro.2012.10.018 23332457 10.1016/j.euroneuro.2012.10.018

[CR16] González-Arias C, Perea G (2025) When the Stars Misfire: Astrocytic Dysfunctions in Major Depressive Disorder. Neurochem Res 50:4 50:231. 10.1007/S11064-025-04483-Y 10.1007/s11064-025-04483-y PMC12255599 40650814

[CR17] Helmut K, Hanisch UK, Noda M, Verkhratsky A (2011) Physiology of microglia. Physiol Rev 91:461–553. 10.1152/PHYSREV.00011.2010 21527731 10.1152/physrev.00011.2010

[CR18] Hernández-Echeagaray E, Vázquez-Roque R, Morales-Medina JC et al (2025) Chlorogenic Acid as a Neuroprotective Agent: Enhancing Plasticity and Promoting Brain Health and Functional Reserve. CNS Neurol Disord Drug Targets 24. 10.2174/0118715273339375250116042441 10.2174/0118715273339375250116042441 40017251

[CR19] Homberg JR, Van Den Akker M, Raasø HS et al (2002) Enhanced motivation to self-administer cocaine is predicted by self‐grooming behaviour and relates to dopamine release in the rat medial prefrontal cortex and amygdala. Eur J Neurosci 15:1542–1550. 10.1046/j.1460-9568.2002.01976.x 12028365 10.1046/j.1460-9568.2002.01976.x

[CR21] Kalueff AV, Tuohimaa P (2005) The grooming analysis algorithm discriminates between different levels of anxiety in rats: potential utility for neurobehavioural stress research. J Neurosci Methods 143:169–177. 10.1016/J.JNEUMETH.2004.10.001 15814150 10.1016/j.jneumeth.2004.10.001

[CR20] Kalueff AV, Stewart AM, Song C et al (2016) Neurobiology of rodent self-grooming and its value for translational neuroscience. Nat Rev Neurosci 17:45–59. 10.1038/nrn.2015.8 26675822 10.1038/nrn.2015.8 PMC4840777

[CR22] Kelly JP, Wrynn AS, Leonard BE (1997a) The olfactory bulbectomized rat as a model of depression: An update. Pharmacol Ther 74:299–316. 10.1016/S0163-7258(97)00004-1 9352586 10.1016/s0163-7258(97)00004-1

[CR23] Kilkenny C, Browne WJ, Cuthill IC et al (2010) Improving Bioscience Research Reporting: The ARRIVE Guidelines for Reporting Animal Research. PLoS Biol 8:e1000412. 10.1371/JOURNAL.PBIO.1000412 20613859 10.1371/journal.pbio.1000412 PMC2893951

[CR24] Kreisel T, Frank MG, Licht T et al (2014) Dynamic microglial alterations underlie stress-induced depressive-like behavior and suppressed neurogenesis. Mol Psychiatry 19:699–709. 10.1038/MP.2013.155 24342992 10.1038/mp.2013.155

[CR25] Leonard BE, Tuite M (1981) Anatomical, Physiological, and Behavioral Aspects of Olfactory Bulbectomy in The Rat. Int Rev Neurobiol 251–286 10.1016/s0074-7742(08)60295-0 7024168

[CR26] Lever C, Burton S, O’Keefe J (2006) Rearing on hind legs, environmental novelty, and the hippocampal formation. Rev Neurosci 17:111–133. 10.1515/REVNEURO.2006.17.1-2.111 16703946 10.1515/revneuro.2006.17.1-2.111

[CR27] Linge R, Pazos Á, Díaz Á (2013) Social isolation differentially affects anxiety and depressive-like responses of bulbectomized mice. Behav Brain Res 245:1–6. 10.1016/j.bbr.2013.01.041 23416113 10.1016/j.bbr.2013.01.041

[CR28] Lombion-Pouthier S, Vandel P, Nezelof S et al (2006) Odor perception in patients with mood disorders. J Affect Disord 90:187–191. 10.1016/J.JAD.2005.11.012 16376994 10.1016/j.jad.2005.11.012

[CR29] Machado DG, Lara MVS, Dobler PB et al (2020) Caffeine prevents neurodegeneration and behavioral alterations in a mice model of agitated depression. Prog Neuropsychopharmacol Biol Psychiatry 98. 10.1016/j.pnpbp.2019.109776 10.1016/j.pnpbp.2019.109776 31707092

[CR30] Malhi GS, Mann JJ (2018) Depression. Lancet 392(10161):2299–2312. 10.1016/S0140-6736(18)31948-2 10.1016/S0140-6736(18)31948-2 30396512

[CR31] McEwen BS, Nasca C, Gray JD (2016) Stress Effects on Neuronal Structure: Hippocampus, Amygdala, and Prefrontal Cortex. Neuropsychopharmacology 41:3–23. 10.1038/NPP.2015.171;TECHMETA 26076834 10.1038/npp.2015.171 PMC4677120

[CR32] McKlveen JM, Morano RL, Fitzgerald M et al (2016) Chronic Stress Increases Prefrontal Inhibition: A Mechanism for Stress-Induced Prefrontal Dysfunction. Biol Psychiatry 80:754–764. 10.1016/j.biopsych.2016.03.2101 27241140 10.1016/j.biopsych.2016.03.2101 PMC5629635

[CR33] Miguel-Hidalgo JJ, Baucom C, Dilley G et al (2000) Glial fibrillary acidic protein immunoreactivity in the prefrontal cortex distinguishes younger from older adults in major depressive disorder. Biol Psychiatry 48:861–873. 10.1016/S0006-3223(00)00999-9 11063981 10.1016/s0006-3223(00)00999-9

[CR34] Morales-Medina JC, Dumont Y, Benoit CE et al (2012) Role of neuropeptide y Y 1 and Y 2 receptors on behavioral despair in a rat model of depression with co-morbid anxiety. Neuropharmacology 62:200–208. 10.1016/j.neuropharm.2011.06.030 21803058 10.1016/j.neuropharm.2011.06.030

[CR36] Morales-Medina JC, Juarez I, Iannitti T, Flores G (2013a) Olfactory bulbectomy induces neuronal rearrangement in the entorhinal cortex in the rat. J Chem Neuroanat 52:80–86. 10.1016/j.jchemneu.2013.07.001 23871725 10.1016/j.jchemneu.2013.07.001

[CR37] Morales-Medina JC, Juarez I, Venancio-García E et al (2013b) Impaired structural hippocampal plasticity is associated with emotional and memory deficits in the olfactory bulbectomized rat. Neuroscience 236:233–243. 10.1016/j.neuroscience.2013.01.037 23357118 10.1016/j.neuroscience.2013.01.037

[CR35] Morales-Medina JC, Iannitti T, Freeman A, Caldwell HK (2017) The olfactory bulbectomized rat as a model of depression: The hippocampal pathway. Behav Brain Res 317:562–575. 10.1016/j.bbr.2016.09.029 27633561 10.1016/j.bbr.2016.09.029

[CR38] Munshi S, Loh MK, Ferrara N et al (2020) Repeated stress induces a pro-inflammatory state, increases amygdala neuronal and microglial activation, and causes anxiety in adult male rats. Brain Behav Immun 84:180–199. 10.1016/j.bbi.2019.11.023 31785394 10.1016/j.bbi.2019.11.023 PMC7010555

[CR39] Nedogreeva OA, Stepanichev MY, Gulyaeva NV (2020) Removal of the Olfactory Bulbs in Mice Leads to Changes in Affective Behavior. Neurosci Behav Physiol 2020 50:7. 10.1007/S11055-020-00982-3

[CR40] Nimmerjahn A, Kirchhoff F, Helmchen F (2005) Resting microglial cells are highly dynamic surveillants of brain parenchyma in vivo. Science 308:1314–1318. 10.1126/SCIENCE.1110647 15831717 10.1126/science.1110647

[CR41] Pekny M, Pekna M (2014) Astrocyte reactivity and reactive astrogliosis: costs and benefits. Physiol Rev 94:1077–1098. 10.1152/PHYSREV.00041.2013 25287860 10.1152/physrev.00041.2013

[CR42] Pekny M, Pekna M, Messing A et al (2016) Astrocytes: a central element in neurological diseases. Acta Neuropathol 131:323–345. 10.1007/S00401-015-1513-1 26671410 10.1007/s00401-015-1513-1

[CR43] Perry VH, Holmes C (2014) Microglial priming in neurodegenerative disease. Nat Rev Neurol 10:217–224. 10.1038/NRNEUROL.2014.38 24638131 10.1038/nrneurol.2014.38

[CR44] Rahimian R, Perlman K, Fakhfouri G et al (2024) Proteomic evidence of depression-associated astrocytic dysfunction in the human male olfactory bulb. Brain Behav Immun 122:110–121. 10.1016/j.bbi.2024.08.016 39128570 10.1016/j.bbi.2024.08.016

[CR46] Rajkowska G, Stockmeier C (2013) Astrocyte pathology in major depressive disorder: insights from human postmortem brain tissue. Curr Drug Targets 14:1225–1236. 10.2174/13894501113149990156 23469922 10.2174/13894501113149990156 PMC3799810

[CR45] Rajkowska G, Miguel-Hidalgo JJ, Wei J et al (1999) Morphometric evidence for neuronal and glial prefrontal cell pathology in major depression. Biol Psychiatry 45:1085–1098. 10.1016/S0006-3223(99)00041-4 10331101 10.1016/s0006-3223(99)00041-4

[CR47] Rodrigues J, Rocha MI, Teixeira F et al (2023) Structural, functional and behavioral impact of allergic rhinitis on olfactory pathway and prefrontal cortex. Physiol Behav 265:114171. 10.1016/J.PHYSBEH.2023.114171 36965572 10.1016/j.physbeh.2023.114171

[CR48] Roozendaal B, McEwen BS, Chattarji S (2009) Stress, memory and the amygdala. Nat Rev Neurosci 10:423–433. 10.1038/NRN2651 19469026 10.1038/nrn2651

[CR49] Schneider CA, Rasband WS, Eliceiri KW (2012) NIH Image to ImageJ: 25 years of image analysis. Nat Methods 9:7 9:671–675 10.1038/nmeth.2089 10.1038/nmeth.2089 PMC5554542 22930834

[CR50] Scuderi C, Verkhratsky A, Parpura V, Li B (2021) Neuroglia in Psychiatric Disorders. Adv Neurobiol 26:3–19. 10.1007/978-3-030-77375-5_1 34888828 10.1007/978-3-030-77375-5_1 PMC9063382

[CR51] Si X, Miguel-Hidalgo JJ, O’Dwyer G et al (2004) Age-Dependent Reductions in the Level of Glial Fibrillary Acidic Protein in the Prefrontal Cortex in Major Depression. Neuropsychopharmacology 29:2088–2096. 10.1038/sj.npp.1300525 15238995 10.1038/sj.npp.1300525 PMC3146059

[CR52] Smolinsky AN, Bergner CL, LaPorte JL, Kalueff AV (2009) Analysis of Grooming Behavior and Its Utility in Studying Animal Stress, Anxiety, and Depression. Neuromethods 42:21–36. 10.1007/978-1-60761-303-9_2

[CR53] Sofroniew MV, Vinters HV (2010) Astrocytes: biology and pathology. Acta Neuropathol 119:7–35. 10.1007/S00401-009-0619-8 20012068 10.1007/s00401-009-0619-8 PMC2799634

[CR54] Song C, Leonard BE (2005) The olfactory bulbectomised rat as a model of depression. Neurosci Biobehav Rev 29:627–647. 10.1016/j.neubiorev.2005.03.010 15925697 10.1016/j.neubiorev.2005.03.010

[CR55] Sturman O, Germain PL, Bohacek J (2018) Exploratory rearing: a context- and stress-sensitive behavior recorded in the open-field test. Stress 21:443–452. 10.1080/10253890.2018.1438405 29451062 10.1080/10253890.2018.1438405

[CR56] Torres-Platas SG, Hercher C, Davoli MA et al (2011) Astrocytic Hypertrophy in Anterior Cingulate White Matter of Depressed Suicides. Neuropsychopharmacology 36:2650–2658. 10.1038/npp.2011.154 21814185 10.1038/npp.2011.154 PMC3230489

[CR57] Tynan RJ, Naicker S, Hinwood M et al (2010) Chronic stress alters the density and morphology of microglia in a subset of stress-responsive brain regions. Brain Behav Immun 24:1058–1068. 10.1016/j.bbi.2010.02.001 20153418 10.1016/j.bbi.2010.02.001

[CR58] Walsh RN, Cummins RA (1976) The open-field test: a critical review. Psychol Bull 83(3):482–504 17582919

[CR59] WHO (2025) Depressive disorder (depression). https://www.who.int/news-room/fact-sheets/detail/depression. Accessed 6 Jan 2026

[CR60] Wohleb ES, Franklin T, Iwata M, Duman RS (2016) Integrating neuroimmune systems in the neurobiology of depression. Nat Rev Neurosci 17:497–511. 10.1038/NRN.2016.69 27277867 10.1038/nrn.2016.69

[CR61] Wohleb ES, Terwilliger R, Duman CH, Duman RS (2018) Stress-Induced Neuronal Colony Stimulating Factor 1 Provokes Microglia-Mediated Neuronal Remodeling and Depressive-like Behavior. Biol Psychiatry 83:38–49. 10.1016/j.biopsych.2017.05.026 28697890 10.1016/j.biopsych.2017.05.026 PMC6506225

[CR62] Young RA, Shin JD, Guo Z, Jadhav SP (2024) Hippocampal-prefrontal communication subspaces align with behavioral and network patterns in a spatial memory task. Preprint. bioRxiv 2024.07.08.601617. 10.1101/2024.07.08.601617 10.1523/ENEURO.0336-24.2025 PMC12463551 40854711

[CR63] Zhang AY, Elias E, White AG et al (2025) Microglia reactivity is brain region and sex specific in the context of chronic stress. Sci Rep 2025 15(1):33285. 10.1038/s41598-025-18000-2 10.1038/s41598-025-18000-2 PMC12475140 41006452

