# Supplementary material for: Early-stage olfactory bulbectomy induces hyperlocomotion with increased astrocyte and microglial density in the prefrontal cortex of male rats
Source: Brain Struct Funct. 2026 Jun 13;231(6):86. doi: 10.1007/s00429-026-03139-z (PMC13264544; doi:10.1007/s00429-026-03139-z)
Supplement: Supplementary file 1 — Supplementary Material 1 [file 429_2026_3139_MOESM1_ESM.docx]

| **Supplementary Table 1** | | | | | | | | |
| --- | --- | --- | --- | --- | --- | --- | --- | --- |
| **Fig 2** | Sham | | | OBX | | | **Effect size (Cohen’s d)** | Graph |
|  | Mean | SD | n | Mean | SD | n |  |  |
| Distance traveled in center | 66.9235 | 56.97386994 | 10 | 128.81993 | 178.4113962 | 10 | 0.46 | 2c |
| Grooming events | 7 | 4.447221355 | 10 | 8.4 | 4.695151163 | 10 | 0.31 | 2d |
| Rearing events | 30.6 | 6.883151733 | 10 | 38.1 | 13.55195763 | 10 | 0.7 | 2e |

| **Supplementary Table 2** | | | | | | | | | |
| --- | --- | --- | --- | --- | --- | --- | --- | --- | --- |
| **Fig 3** | | Sham | | | OBX | | | **Effect size (Cohen’s d)** | Graph |
|  |  | Mean | SD | n | Mean | SD | n |  |  |
| astrocytic branch length | | 378.15 | 24.78916654 | 10 | 370.1 | 28.53633162 | 10 | 0.3 | 3c |
| microglia branch length | | 412.35 | 44.37407276 | 10 | 402.5 | 46.32433965 | 10 | 0.22 | 3j |
| ANOVA table | | | | | | | | | |
|  | | F (DFn, DFd) | P value | **η²p** | Graph | F (DFn, DFd) | P value | **η²p** | Graph |
| radial complexity | Interaction | F (9, 180) = 0.3693 | P=0.9484 | 0.018 | 3d | F (11, 216) = 0.5767 | P=0.8467 | 0.029 | 3k |
|  | Distance | F (9, 180) = 563.9 | P<0.0001 | 0.966 |  | F (11, 216) = 639.6 | P<0.0001 | 0.97 |  |
|  | Group | F (1, 180) = 1.064 | P=0.3036 | 0.006 |  | F (1, 216) = 1.199 | P=0.2747 | 0.006 |  |
| length per branching order | Interaction | F (5, 108) = 0.2794 | P=0.9235 | 0.013 | 3e | F (5, 108) = 0.8547 | P=0.5142 | 0.038 | 3l |
|  | Order | F (5, 108) = 584.5 | P<0.0001 | 0.964 |  | F (5, 108) = 505.4 | P<0.0001 | 0.96 |  |
|  | Group | F (1, 108) = 0.3950 | P=0.5310 | 0.004 |  | F (1, 108) = 0.5268 | P=0.4695 | 0.005 |  |

| **Supplementary Table 3** | | | | | | | | | |
| --- | --- | --- | --- | --- | --- | --- | --- | --- | --- |
| **Fig 4** | | Sham | | | OBX | | | **Effect size (Cohen’s d)** | Graph |
|  |  | Mean | SD | n | Mean | SD | n |  |  |
| number of astrocytes | | 43.12 | 7.006156023 | 10 | 40.52 | 6.761459409 | 10 | 0.38 | 4a |
| astrocytic branch length | | 351.15 | 54.08329481 | 10 | 347.25 | 71.07595702 | 10 | 0.06 | 4c |
| number of microglia | | 40.86 | 3.021478667 | 10 | 40.52 | 4.361651064 | 10 | 0.09 | 4h |
| microglia branch length | | 406.75 | 36.44344934 | 10 | 417.55 | 22.62305854 | 10 | 0.36 | 4j |
| ANOVA table | | | | | | | | | |
|  | | F (DFn, DFd) | P value | **η²p** | Graph | F (DFn, DFd) | P value | **η²p** | Graph |
| distance from bregma distribution | Interaction | F (4, 90) = 0.9868 | P=0.4189 | 0.042 | 4b | F (4, 90) = 0.1534 | P=0.9610 | 0.007 | 4i |
|  | Distance | F (4, 90) = 1.072 | P=0.3754 | 0.046 |  | F (4, 90) = 0.2667 | P=0.8987 | 0.012 |  |
|  | Group | F (1, 90) = 1.887 | P=0.1730 | 0.021 |  | F (1, 90) = 0.1298 | P=0.7194 | 0.001 |  |
| radial complexity | Interaction | F (10, 198) = 0.06736 | P>0.9999 | 0.003 | 4d | F (11, 216) = 0.8894 | P=0.5516 | 0.043 | 4k |
|  | Distance | F (10, 198) = 283.1 | P<0.0001 | 0.935 |  | F (11, 216) = 652.7 | P<0.0001 | 0.971 |  |
|  | Group | F (1, 198) = 0.09609 | P=0.7569 | 0.0005 |  | F (1, 216) = 1.772 | P=0.1846 | 0.008 |  |
| length per branching order | Interaction | F (6, 126) = 0.3105 | P=0.9305 | 0.015 | 4e |  | | | |
|  | Order | F (6, 126) = 278.2 | P<0.0001 | 0.93 |  |  |  |  |  |
|  | Group | F (1, 126) = 0.04804 | P=0.8269 | 0.0004 |  |  |  |  |  |

| **Supplementary Table 4** | | | | | | | | | |
| --- | --- | --- | --- | --- | --- | --- | --- | --- | --- |
| **Fig 5** | | Sham | | | OBX | | | **Effect size (Cohen’s d)** | Graph |
|  |  | Mean | SD | n | Mean | SD | n |  |  |
| number of astrocytes | | 41.38 | 6.152470145 | 10 | 41.02 | 6.090758026 | 10 | 0.06 | 5a |
| astrocytic branch length | | 431.25 | 56.03334821 | 10 | 410.4 | 58.54902979 | 10 | 0.36 | 5c |
| number of microglia | | 49.74 | 5.14505156 | 10 | 47.42222222 | 6.825279807 | 9 | 0.39 | 5h |
| microglia branch length | | 278.15 | 36.33719398 | 10 | 274.7777778 | 40.19336941 | 9 | 0.09 | 5j |
| ANOVA table | | | | | | | | | |
|  | | F (DFn, DFd) | P value | **η²p** | Graph | F (DFn, DFd) | P value | **η²p** | Graph |
| distance from bregma distribution | Interaction | F (4, 90) = 0.5232 | P=0.7189 | 0.023 | 5b | F (4, 85) = 0.1967 | P=0.9395 | 0.009 | 5i |
|  | Distance | F (4, 90) = 2.650 | P=0.0383 | 0.105 |  | F (4, 85) = 0.7559 | P=0.5569 | 0.034 |  |
|  | Group | F (1, 90) = 0.05345 | P=0.8177 | 0.0006 |  | F (1, 85) = 1.573 | P=0.2132 | 0.018 |  |
| radial complexity | Interaction | F (13, 252) = 0.6882 | P=0.7743 | 0.034 | 5d | F (9, 170) = 0.2951 | P=0.9754 | 0.015 | 5k |
|  | Distance | F (13, 252) = 448.4 | P<0.0001 | 0.959 |  | F (9, 170) = 339.5 | P<0.0001 | 0.947 |  |
|  | Group | F (1, 252) = 2.963 | P=0.0864 | 0.012 |  | F (1, 170) = 0.1753 | P=0.6760 | 0.001 |  |
| length per branching order | Interaction | F (5, 108) = 1.681 | P=0.1453 | 0.072 | 5e | F (4, 85) = 1.220 | P=0.3083 | 0.054 | 5l |
|  | Order | F (5, 108) = 426.1 | P<0.0001 | 0.952 |  | F (4, 85) = 324.0 | P<0.0001 | 0.938 |  |
|  | Group | F (1, 108) = 1.538 | P=0.2176 | 0.014 |  | F (1, 85) = 0.08080 | P=0.7769 | 0.001 |  |
